# Relacorilant, a Selective Glucocorticoid Receptor Modulator, Induces Clinical Improvements in Patients With Cushing Syndrome: Results From A Prospective, Open-Label Phase 2 Study

**DOI:** 10.3389/fendo.2021.662865

**Published:** 2021-07-14

**Authors:** Rosario Pivonello, Irina Bancos, Richard A. Feelders, Atil Y. Kargi, Janice M. Kerr, Murray B. Gordon, Cary N. Mariash, Massimo Terzolo, Noel Ellison, Andreas G. Moraitis

**Affiliations:** ^1^ Dipartimento di Medicina Clinica e Chirurgia, Sezione di Endocrinologia, Università Federico II di Napoli, Naples, Italy; ^2^ Department of Internal Medicine, Division of Endocrinology, Diabetes, Metabolism, and Nutrition, Mayo Clinic, Rochester, MN, United States; ^3^ Department of Internal Medicine, Division of Endocrinology, Erasmus Medical Center, Rotterdam, Netherlands; ^4^ Department of Medicine, Division of Endocrinology, Diabetes and Metabolism, University of Miami, Miami, FL, United States; ^5^ Department of Endocrinology, Division of Endocrinology, Metabolism and Diabetes, University of Colorado Denver, Aurora, CO, United States; ^6^ Allegheny Neuroendocrinology Center, Allegheny General Hospital, Pittsburgh, PA, United States; ^7^ Methodist Research Institute, Indiana University School of Medicine, Indianapolis, IN, United States; ^8^ Department of Clinical and Biological Sciences, Internal Medicine 1 – San Luigi Gonzaga Hospital, University of Turin, Orbassano, Italy; ^9^ Biostatistics, Trialwise, Inc, Houston, TX, United States; ^10^ Drug Research and Development, Corcept Therapeutics, Menlo Park, CA, United States

**Keywords:** clinical trial, cortisol, Cushing syndrome, glucocorticoid, hypercortisolism, hyperglycemia, hypertension, relacorilant

## Abstract

**Introduction/Purpose:**

Relacorilant is a selective glucocorticoid receptor modulator (SGRM) with no progesterone receptor activity. We evaluated the efficacy and safety of relacorilant in patients with endogenous Cushing syndrome (CS).

**Materials and Methods:**

A single-arm, open-label, phase 2, dose-finding study with 2 dose groups (NCT02804750, https://clinicaltrials.gov/ct2/show/NCT02804750) was conducted at 19 sites in the U.S. and Europe. Low-dose relacorilant (100-200 mg/d; n = 17) was administered for 12 weeks or high-dose relacorilant (250-400 mg/d; n = 18) for 16 weeks; doses were up-titrated by 50 mg every 4 weeks. Outcome measures included proportion of patients with clinically meaningful changes in hypertension and/or hyperglycemia from baseline to last observed visit. For patients with hypertension, clinical response was defined as a ≥5-mmHg decrease in mean systolic or diastolic blood pressure, measured by a standardized and validated 24-h ABPM. For patients with hyperglycemia, clinical response was defined ad-hoc as ≥0.5% decrease in HbA1c, normalization or ≥50-mg/dL decrease in 2-h plasma glucose value on oral glucose tolerance test, or decrease in daily insulin (≥25%) or sulfonylurea dose (≥50%).

**Results:**

35 adults with CS and hypertension and/or hyperglycemia (impaired glucose tolerance or type 2 diabetes mellitus) were enrolled, of which 34 (24 women/10 men) received treatment and had postbaseline data. In the low-dose group, 5/12 patients (41.7%) with hypertension and 2/13 patients (15.4%) with hyperglycemia achieved response. In the high-dose group, 7/11 patients (63.6%) with hypertension and 6/12 patients (50%) with hyperglycemia achieved response. Common (≥20%) adverse events included back pain, headache, peripheral edema, nausea, pain at extremities, diarrhea, and dizziness. No drug-induced vaginal bleeding or hypokalemia occurred.

**Conclusions:**

The SGRM relacorilant provided clinical benefit to patients with CS without undesirable antiprogesterone effects or drug-induced hypokalemia.

## Introduction

Endogenous hypercortisolism (Cushing syndrome [CS]) is a complex, multisystem endocrine disorder characterized by cortisol excess and is frequently associated with hypertension and hyperglycemia (including impaired glucose tolerance/type 2 diabetes mellitus [IGT/T2DM]) (1, 2). CS, especially if untreated, is associated with increased cardiovascular-related mortality and multiple morbidities beyond hypertension and hyperglycemia, including visceral obesity, dyslipidemia, liver steatosis, osteoporosis, hypercoagulopathy, susceptibility to infection, neuropsychiatric disorders, and reproductive and sexual disturbances (2–11).

Medical therapies, including pituitary-targeting agents, steroid synthesis inhibitors, and glucocorticoid receptor [GR] antagonists, are a treatment option for patients who are not candidates for surgery, and for patients with persistent or recurrent hypercortisolism after surgery who are unsuitable for, or unwilling to undergo, additional surgical procedures (12–15).

The competitive GR and progesterone antagonist mifepristone was approved by the Food and Drug Administration in 2012 to control hyperglycemia in patients with endogenous CS who have IGT or T2DM. The clinical benefit of mifepristone in patients with CS was shown in an open-label, phase 3 trial, SEISMIC (Study of the Efficacy and Safety of Mifepristone in the Treatment of Endogenous Cushing Syndrome) (16). Serum cortisol and ACTH levels may rise in response to GR antagonism with mifepristone (15, 17, 18). In patients with CS, further increases in cortisol in turn can lead to stimulation of the mineralocorticoid receptor, resulting in adverse events such as hypertension and hypokalemia (15, 18, 19). Because of its mechanism of action, clinical and metabolic parameters, rather than cortisol levels, must be monitored in patients during mifepristone treatment for assessment of efficacy (18).

Relacorilant (CORT125134, Corcept Therapeutics, Menlo Park, CA) is an investigational, highly selective GR modulator that competitively antagonizes cortisol activity (20). Unlike mifepristone, relacorilant does not bind to the progesterone receptor (20). Given its binding affinity profile, it was hypothesized that relacorilant would provide patients with endogenous CS the treatment benefits of cortisol modulation, but without the unwanted effects of progesterone receptor antagonism (e.g., induction of abortion, progesterone receptor modulator-associated endometrial changes and irregular vaginal bleeding in women).

This dose-finding study was designed to assess the efficacy and safety of relacorilant in patients with endogenous hypercortisolism due to either excess ACTH secretion (from a pituitary or other ectopic tumor) or autonomous adrenal cortisol secretion, as well as to inform phase 3 study design. Because GR antagonism lowers cortisol activity, but not cortisol levels (16), an improvement in clinical manifestations of hypercortisolism were evaluated as assessments of efficacy with relacorilant. This study assessed the impact of reduced cortisol activity on blood pressure and parameters of blood glucose control following treatment with relacorilant in patients with CS. Additional secondary and exploratory outcome measures were also assessed.

## Materials and Methods

### Study Design

**Figure 1:**
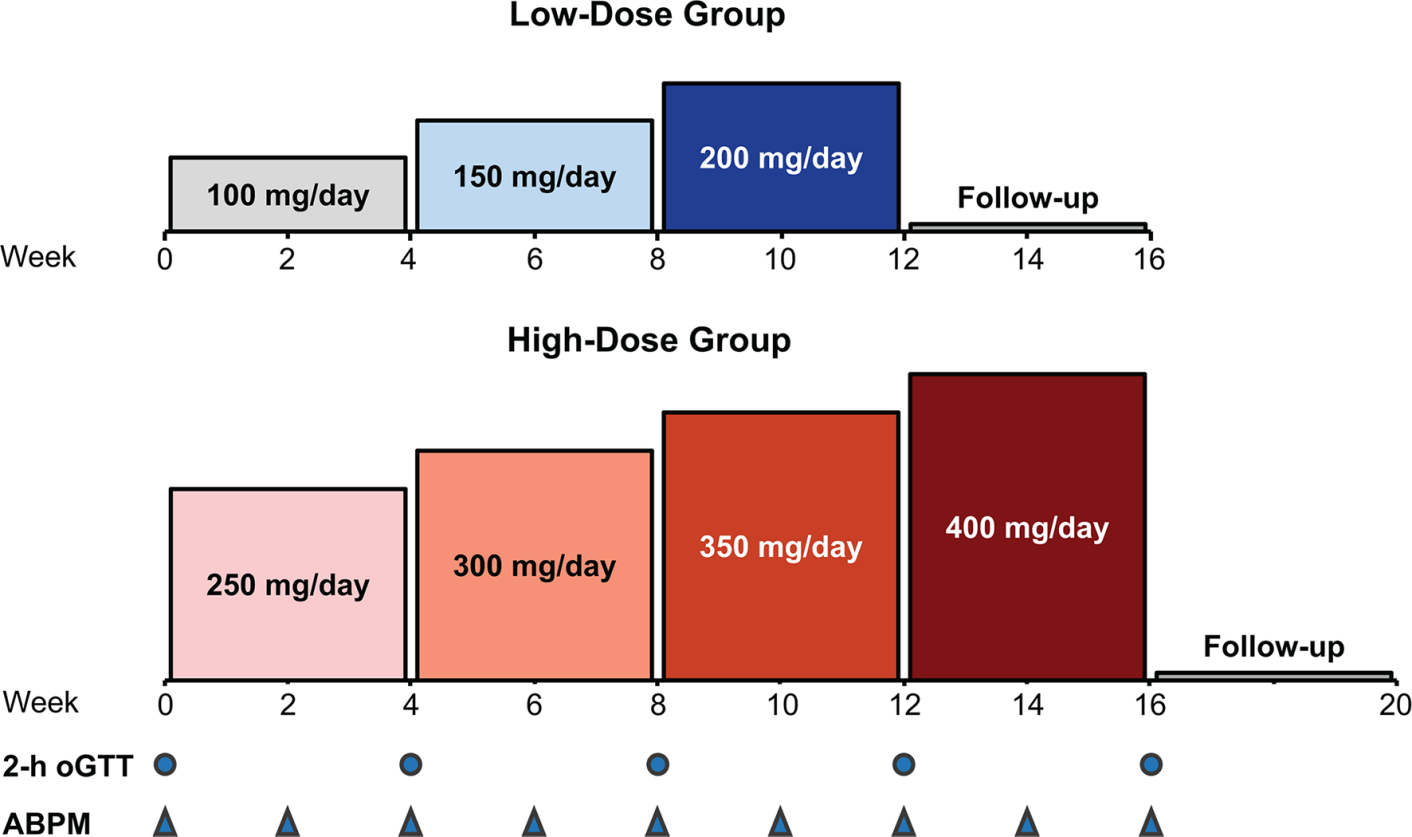
Study design: dose escalation in the low- and high-dose groups. ABPM, ambulatory blood pressure monitoring; oGTT, oral glucose tolerance test.

This phase 2, multicenter, single-arm, open-label, dose-finding study (NCT02804750) assessed the efficacy and safety of relacorilant using 2 dose groups: a low-dose group and a high-dose group. The dose in each group was increased by 50 mg every 4 weeks (**Figure 1**). The low-dose group received a starting relacorilant dose of 100 mg/d, followed by 150 mg/d and then 200 mg/d. The high-dose group began with a starting relacorilant dose of 250 mg/d, followed by 300 mg/d, 350 mg/d, and 400 mg/d. Dose reductions were permitted at the discretion of the investigator for safety/tolerability. Enrolment in the high-dose group began after completion of enrollment of the low-dose group.

The study was approved by the institutional review board at each study center and conducted in accordance with the World Medical Association Declaration of Helsinki and the International Council on Harmonisation Good Clinical Practice guidelines. All patients provided written informed consent. The study was conducted at 19 centers in the United States, Italy, United Kingdom, Hungary, and The Netherlands between February 2017 and September 2018.

### Patients

Adult patients aged 18 to 80 years with a diagnosis of endogenous CS and requiring medical treatment (i.e., those for whom surgery or radiation is contraindicated or has been refused) were eligible for the study. These patients must have met at least 2 of the following biochemical criteria based on the Endocrine Society Guidelines (Nieman 2008): (1) a 24-h urinary free cortisol (UFC) above the upper limit of normal (ULN) (50 µg/24 h [138 nmol/d]) on at least 2 collections, (2) a late-night salivary cortisol (LNSC) value above ULN (10-11 PM normal range, ≤0.09 µg/dL [2.5 nmol/L]) on at least 2 collections, or (3) a lack of cortisol suppression (>1.8 µg/dL serum cortisol [49.7 nmol/L]) on either the overnight 1-mg or 48-h 2-mg dexamethasone suppression test (DST) (1, 21). In addition, patients were also required to have at least 2 clinical signs or symptoms of hypercortisolism: a Cushingoid appearance (moon facies, dorsocervical fat pad, and/or facial plethora), increased body weight or central obesity, proximal muscle weakness, low bone mass (dual energy X-ray absorptiometry T < -1.0), psychiatric symptoms (including depression or psychosis), easy bruising, or skin changes (hirsutism, violaceous striae, and/or acne) (1).

Patients with adrenal adenomas were also eligible if they met the following criteria for autonomous cortisol secretion based on the European Society of Endocrinology/European Network for the Study of Adrenal Tumors Guidelines (Fassnacht 2016): unilateral or bilateral adrenal disease, lack of cortisol suppression (>5 µg/dL [>138 nmol/L] serum cortisol) on either 1-mg or 48-h 2-mg DST, low or suppressed ACTH (<10 pg/mL [<2.2 pmol/L]), and presence of at least 2 comorbidities potentially related to cortisol excess (e.g., T2DM, hypertension, obesity, osteoporosis) (21).

Because changes in blood pressure and glucose tolerance served as key efficacy endpoints, patients included in the study were required to have uncontrolled hypertension (hypertension cohort), and/or IGT or T2DM (hyperglycemia cohort). Uncontrolled hypertension was defined as a mean systolic blood pressure (SBP) of ≥130 mmHg and/or mean diastolic blood pressure (DBP) of ≥85 mmHg using 24-h ambulatory blood pressure monitoring (ABPM) based on the European Society of Hypertension Position Paper (O’Brien 2013) (22). IGT was defined as a 2-h oral glucose tolerance test (oGTT) plasma glucose value of 140-199 mg/dL (7.8-11.0 mmol/L) after 75 g of glucose, while T2DM was defined as a fasting plasma glucose >126 mg/dL (7.0 mmol/L) or a 2-h plasma glucose ≥200 mg/dL (11.1 mmol/L) after a 75-g oGTT (23). Patients on antidiabetic or antihypertensive medications were allowed in the study if their doses were stable and had not been changed for at least 1 month before the baseline assessment. Patients receiving medications for CS underwent an appropriate washout duration before baseline assessments were performed (e.g., 1 month for adrenostatic medications such as metyrapone and ketoconazole; 2 months for long-acting somatostatin analogs and dopamine agonists; 1 month for short-acting somatostatin analogs; and 6 weeks for mifepristone).

Patients with SBP >170 or DBP >110 mmHg, glycated hemoglobin (HbA1c) >12%, uncontrolled hypothyroidism or hyperthyroidism, or uncorrected hypokalemia (<3.5 mEq/L) were excluded. Concurrent use of other medications for CS was not allowed in the study. Patients who had undergone radiation therapy for CS within 1 year of screening were also excluded.

### Assessments

#### Patients With Hypertension

Assessment of hypertension was performed using standardized, validated 24-h ABPM to allow for an accurate measurement (22, 24). A clinically significant response was defined as a decrease of ≥5 mmHg in either mean 24-h SBP or DBP from baseline without an increase in dosage of concurrent antihypertensive medication or initiation of additional antihypertensive medication during the treatment period.

#### Patients With Hyperglycemia

To assess glucose tolerance, a 2-h oGTT was administered and HbA1c was evaluated in all patients with hyperglycemia. Clinically meaningful response was defined ad-hoc as one of the following: a decrease in HbA1c of ≥0.5% from baseline, normalization of the 2-h plasma glucose value on the oGTT(<140 mg/dL [<7.8 mmol/L]) or a decrease in the 2-h plasma glucose value on the oGTT by ≥50 mg/dL (≥2.8 mmol/L) from baseline, or a decrease in the total daily insulin dose by ≥25% or a decrease in the daily sulfonylurea dose by ≥50% from baseline. Based on the criterion used in the pivotal study of mifepristone, SEISMIC (16), the hyperglycemia response was initially defined as a decrease of ≥25% from baseline in the area under the concentration-time curve for glucose (AUC_glucose_), without an increase in dosage of concurrent antidiabetes medication or additional antidiabetes medication during the treatment period. However, unlike SEISMIC, a substantial portion of the patients enrolled in the hyperglycemia group in the current study had IGT rather than overt diabetes, and the patients with diabetes had much better glycemic control (mean HbA1c was 6.6%, compared with 7.4% in SEISMIC) (16). Thus, a ≥25% decrease in the total AUC_glucose_ from baseline would not be suitable to detect clinically meaningful improvements in these patients.

### Secondary and Exploratory Endpoints

Considering the numerous additional comorbidities and clinical complications associated with CS, including obesity, impaired glucose metabolism and insulin resistance, liver steatosis, osteoporosis, immune disorders, thrombosis, and neuropsychiatric diseases (2, 3, 5, 10, 11, 25), the effect of relacorilant on several secondary and exploratory endpoints was also assessed. These included changes in body weight; serum fructosamine an indicator of glucose metabolism control (26); homeostatic model assessment of insulin resistance (HOMA-IR); liver function tests; serum osteocalcin, a marker of bone metabolism (25); eosinophils; activated partial thromboplastin time (aPTT), Factor VIII, and platelet count for assessment of coagulation; quality of life (QoL) as assessed using the Cushing QoL Questionnaire (27); depressive symptoms as assessed using the Beck Depression Inventory (BDI-II) (28); and cognitive function evaluated using the Trail Making Test (Parts A and B) (29). Hormone changes were assessed using plasma ACTH, and cortisol (24-h UFC, LNSC, and serum cortisol). Treatment-emergent AEs (TEAEs) were assessed at every visit for safety. Potassium levels were monitored, particularly for the emergence of hypokalemia.

Blood, urine, and saliva samples were collected and analyzed centrally (by Q^2^ Solutions Laboratories, Valencia, CA; Quest Diagnostics Nichol Institute, San Juan Capistrano, CA was used for hormonal testing only). AUC_glucose_ was calculated based on results of the 2-h oGTTs. Plasma glucose and insulin values from the baseline oGTT assessment (pre-glucose drink) were used to calculate HOMA-IR (30). Urinary cortisol (normal range, 4-50 µg/24h [11-138 nmol/d]) was measured by tandem mass spectrometry (MS/MS). Serum cortisol (8-10 AM normal range, 4.6-20.6 µg/dL [127-567 nmol/L]) levels and LNSC levels (10-11 PM normal range, ≤0.09 µg/dL [≤ 2.5 nmol/L]), were measured by liquid chromatography LC-MS/MS. Plasma ACTH (7-10 AM normal range, 6-50 pg/mL [1.3-11.1 pmol/L]) was measured with an immunochemiluminescent assay.

### Statistical Analysis

No formal sample size calculation was performed. A sample size of 30 patients (15 per dose group) was deemed sufficient for a preliminary evaluation of the efficacy and safety of relacorilant and to establish estimates of efficacy for future evaluation of the study drug. All patients who received at least one dose of study medication were included in the safety analysis. All patients who received at least one dose of study medication and had postbaseline data were included in the efficacy population. Because of the small population in the clinical responder analyses, additional exclusions for major protocol violations were applied to specific visits or outcomes for patients in the hypertension and hyperglycemia groups, rather than excluding the patient entirely.

The key efficacy endpoints (hypertension and hyperglycemia responders) were analyzed by dose group at Week 12 or early termination (ET) for the low-dose group and Week 16 or ET for the high-dose group, and summarized as last observed. The number and percentage of patients considered responders was presented along with the 95% exact binomial two-sided confidence intervals (Clopper-Pearson). Because there was no comparator group, statistical significance was determined if the lower limit of the 95% exact binomial CIs for the responder rate was >20%. This threshold for response was selected based on the similar threshold used in the previously reported SEISMIC study (16). SAS statistical software version 9.4 or higher (SAS Institute, Cary, NC) was used.

Descriptive statistics were used to summarize changes from baseline in secondary and exploratory outcomes, including weight, fructosamine, HOMA-IR, liver function tests, serum osteocalcin, absolute eosinophils, aPTT, Factor VIII, platelet count, QoL, depressive symptoms, cognitive function, and TEAEs. Means or medians and Wilcoxon signed-rank *p*-values were calculated to assess change from baseline for exploratory outcomes (fructosamine, HOMA-IR, osteocalcin, absolute eosinophils, coagulation parameters, QoL, depressive symptoms, cognitive function, plasma ACTH, 24-h UFC, LNSC, and serum cortisol changes).

## Results

### Patients

**Figure 2:**
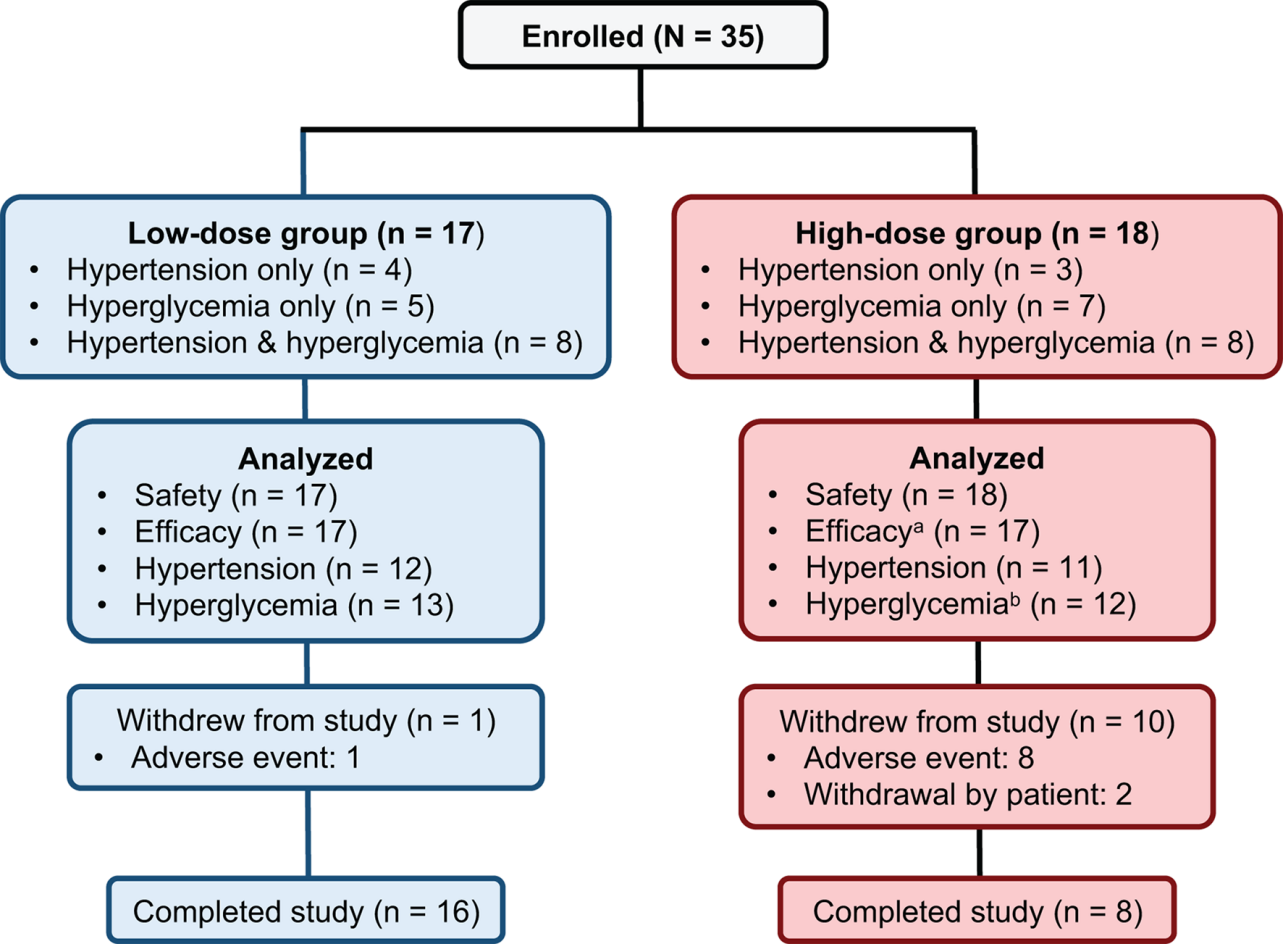
Patient disposition. ^a^One patient in the high-dose group did not have any postbaseline data and was excluded from the efficacy population. ^b^Two patients in the high-dose hyperglycemia group did not have any postbaseline efficacy data while on study drug and were excluded from the hyperglycemia population.

Thirty-five patients meeting the inclusion criteria were enrolled. All 35 received at least 1 treatment dose and were included in the safety population. Seventeen patients were enrolled in the low-dose group and 18 patients were enrolled in the high-dose group. One patient did not have any postbaseline data and was excluded from the efficacy population (n = 34) (**Figure 2**).

Baseline characteristics of the efficacy population (70.6% female [12/24 premenopausal], mean ± SD age of 48.2 ± 13.33 years) are shown in **Tables 1** and **2**. Twenty-seven patients (79.4%) were diagnosed with ACTH-dependent CS, and 7 (20.6%) were diagnosed with ACTH-independent CS (adrenal adenoma). Of the patients with ACTH-dependent CS, 4 were diagnosed with ectopic ACTH secretion. Among the total 34 patients, 47.1% (n = 16; 8 low-dose/8 high-dose group) were diagnosed with both hyperglycemia and hypertension, 32.4% (n = 11; 5 low-dose/6 high-dose group) were diagnosed with hyperglycemia only, and 20.6% (n = 7; 4 low-dose/3 high-dose group) were diagnosed with hypertension only. Among patients in the hypertension analysis group (n = 23), mean 24-h blood pressure (SBP/DBP) at baseline was 138.3/87.0 mmHg. Mean HbA1c at baseline among patients in the hyperglycemia analysis group (n = 27) was 6.6%. Among patients in the hyperglycemia analysis group, 37.0% (10/27) had IGT and 63.0% (17/27) had T2DM.

**Table 1:** Demographic and clinical characteristics at baseline (efficacy population, n = 34).

	Overall population (n = 34)
Age, years (mean ± SD)	48.2 ± 13.33
Female sex, n (%)	24 (70.6)
White race, n (%)	34 (100)
Ethnicity, n (%)	
Hispanic or Latino	3 (8.8)
Not Hispanic or Latino	31 (91.2)
Weight, kg (mean ± SD)	97.8 ± 27.41
BMI, kg/m^2^ (mean ± SD)	35.6 ± 9.75
Etiology of CS, n (%)	
ACTH-dependent: CD or ectopic, n (%)	27 (79.4)
ACTH, pmol/L (median [min, max])	14.0 (6.0, 28)
24-h UFC, nmol/d (median [min, max])	357.4 (83.4, 2823.8)
Adrenal adenoma (unilateral), n (%)	7 (20.6)
ACTH, pmol/L (median [min, max])	1.0 (1.0, 1.6)
24-h UFC, nmol/d (median [min, max])^a^	156.1 (56.9, 2341.0)
Osteocalcin, µg/L, (mean ± SD)^b^	11.0 ± 7.55
Analysis cohorts	
Hypertension, n (%)	23 (67.6)
24-h SBP, mmHg (mean ± SD)	138.3 ± 8.59
24-h DBP, mmHg (mean ± SD)	87.0 ± 5.65
Hyperglycemia, n (%)	27 (79.4)
IGT, n (%)	10 (37.0)
T2DM, n (%)	17 (63.0)
Fructosamine, µmol/L (mean ± SD)	225.3 ± 29.50
Fasting plasma glucose, mmol/L (mean ± SD)	6.6 ± 1.89
2-h oGTT plasma glucose, mmol/L (mean ± SD)	12.7 ± 4.08
AUC_glucose_, h•mmol/L (mean ± SD)	26.2 ± 8.05
HbA1c, % (mean ± SD)	6.6 ± 1.26

To convert values of glucose to mg/dL, divide by 0.0555; UFC to µg/24 h, divide by 2.76.

a Includes one patient with an adrenal adenoma who had a baseline 24-h UFC of 2,341 nmol/d (848.2 µg/d).

b Osteocalcin n = 33.

ACTH, adrenocorticotropic hormone; AUC, area under the curve; BMI, body mass index; CD, Cushing disease; CS, Cushing syndrome; DBP, diastolic blood pressure; HbA1c, glycated hemoglobin; IGT, impaired glucose tolerance; oGTT, oral glucose tolerance test; SBP, systolic blood pressure; SD, standard deviation; T2DM, type 2 diabetes mellitus; UFC, urinary free cortisol.

**Table 2:** Median biochemistry at baseline (efficacy population, n = 34).

	Low-dose group (n = 17)	High-dose group (n = 17)	Overall population (n = 34)
ACTH, pmol/L (min, max)	9.8 (1.0, 25.3)	14.0 (1.0, 28)	10.8 (1.0, 28.0)
24-h UFC, nmol/d (min, max)	357.4 (122.0, 2823.8)	251.6 (56.9, 2341.0)	357.0 (56.9, 2823.8)
LNSC, nmol/L (min, max)	7.2 (1.2, 36.5)	5.4 (2.1, 32.7)	6.6 (1.2, 36.5)
Serum cortisol, nmol/L (min, max)	483.0 (141.0, 748.0)	431.0 (235.0, 1352.0)	459.5 (141.0, 1352.0)

To convert values of ACTH to pg/mL, divide by 0.22; UFC to µg/24 h, divide by 2.76; LNSC and serum cortisol to µg/dL, divide by 27.6.

ACTH, adrenocorticotropic hormone; LNSC, late-night salivary cortisol; SD, standard deviation; UFC, urinary free cortisol.

### Key Efficacy Analyses

Among patients in the hypertension analysis population (n = 23), 5 of 12 patients (41.7%, 95% CI 15.17, 72.33) in the low-dose group and 7 of 11 patients (63.6%, 95% CI 30.79, 89.07) in the high-dose group were responders at their last observed blood pressure measurement (**Table 3**).

**Table 3:** Summary of responder analysis in patients with hypertension (n = 23) and hyperglycemia (n = 25) at last observation.

	Low-dose group		High-dose group	
	Responder, n/N (%)	95% CI	Responder, n/N (%)	95% CI
Hypertension^a^	5/12 (41.7)	15.17, 72.33	7/11 (63.6)	30.79, 89.07
Hyperglycemia^b^	2/13 (15.4)	1.92, 45.45	6/12 (50.0)	21.09, 78.91

CIs: 95% binomial exact two-sided confidence interval (Clopper-Pearson).

Two patients in the hyperglycemia responder analysis were excluded because they had no postbaseline efficacy data collected while on study drug.

a Response defined as a ≥5 mmHg decrease in mean systolic or diastolic BP from baseline without the use of additional antihypertensive medication or an increase in dosage of a concurrent antihypertensive medication.

b Achieving any of the ad-hoc hyperglycemia response criteria with no increase in HbA1c. Patients who achieve the response criteria but whose HbA1c increases cannot be considered overall responders. Ad-hoc response criteria: a ≥0.5% decrease in HbA1c, normalization (<140 mg/dL [<7.8 mmol/L]) or ≥50-mg/dL (2.8 mmol/L) decrease in 2-h glucose value on oGTT, or decrease in daily insulin (≥25%) or sulfonylurea dose (≥50%).

CI, confidence interval.

**Figure 3:**
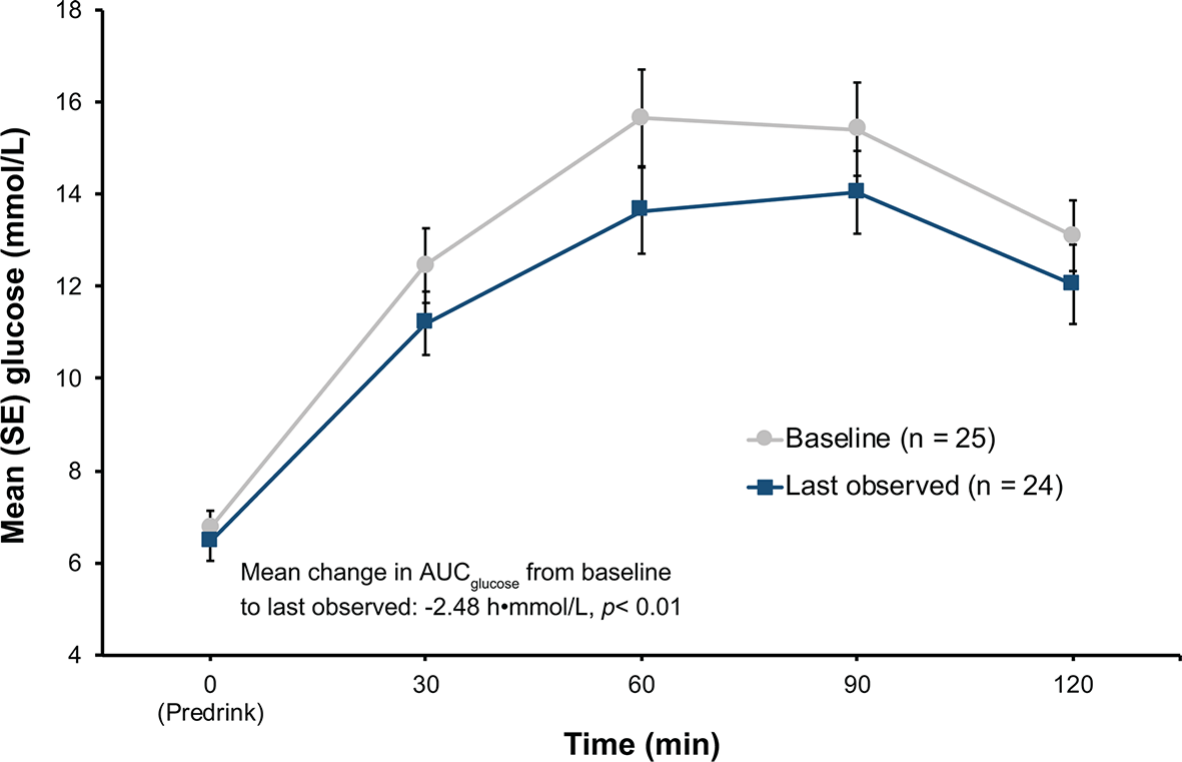
Results of oGTT tests at baseline and at last observation in the hyperglycemia group. *p*-value from Wilcoxon signed-rank. AUC, area under the curve; oGTT, oral glucose tolerance test; SE, standard error.

Among patients in the hyperglycemia analysis population (n = 25), 2 of the 13 patients (15.4%, 95% CI 1.92%, 45.45) in the low-dose group and 6 of the 12 patients (50.0%, CI 21.09%, 78.91%) in the high-dose group met the ad-hoc hyperglycemia response at the last observed visit (**Table 3**). Additionally, when both the low-dose and high-dose groups were combined, plasma glucose levels during oGTT decreased (**Figure 3**), leading to a statistically significant decrease in mean AUC_glucose_ from baseline to the last observed visit (-2.48 h•mmol/L, *p* < 0.01).

### Secondary and Exploratory Efficacy Analyses

#### Changes in Weight

In the efficacy population, the median weight at baseline was similar among patients in the low-dose (85.5 kg) and high-dose (91.1 kg) group. During the study, 7 of 17 patients (41.2%) in the low-dose group and 10 of 16 patients (62.5%) in the high-dose group lost weight. Among the patients who lost weight during the study, the median (min, max) changes from baseline to last observed visit were -2.0 kg (-2.5, -0.4) and -3.0 kg (-15.1, -1.1) for the low-dose and high-dose groups, respectively. Among all patients, the median weight change was 0.50 kg in the low-dose group and -1.75 kg in the high-dose group.

#### Additional Exploratory Endpoints

Statistically significant improvements from baseline were observed in various exploratory endpoints related to cortisol excess in the efficacy population (**Table 4**). These included improvements in fructosamine (mean change -13.92 µmol/L, *p* = 0.002) among all patients with hyperglycemia. Decreases in the liver function parameters alanine aminotransferase (mean change -10.62 U/L, *p* < 0.001) and aspartate aminotransferase (mean change -4.94 U/L, *p* = 0.001), and increases in serum osteocalcin (mean change 3.00 µg/L, p < 0.01) and eosinophil count (mean change 0.05•10^9^/L, *p* = 0.006) were observed in the overall patient population. The aPTT increased significantly (mean change 1.45 sec, *p* = 0.046) and was accompanied by significant decreases in factor VIII (mean change -18.94%, *p* = 0.022) and platelet counts (mean change -68.82•10^9^/L, *p* < 0.001). Statistically significant improvements from baseline to last observed visit were observed in BDI-II Total score (mean change -3.48, *p* = 0.004), Cushing QoL score (mean change 7.13, *p* = 0.002), and Trail Making Test Part A and Part B (mean changes -4.13 sec, *p* = 0.003 and -24.69 sec, *p* < 0.001, respectively).

**Table 4:** Mean change from baseline to last observation in exploratory secondary endpoints (efficacy population, n = 34).

Parameter	Mean change from baseline to last observation	*p*-value
Fructosamine (μmol/L)	-13.92	0.002
HOMA-IR^a^	-1.58	0.064
ALT (U/L)	-10.62	< 0.001
AST (U/L)	-4.94	0.001
Serum osteocalcin (μg/L)	3.00	< 0.01
Absolute eosinophils (10^9^/L)	0.05	0.006
aPTT (sec)	1.45	0.046
Factor VIII (%)	-18.94	0.022
Platelet count (10^9^/L)	-68.82	< 0.001
BDI-II Total score	-3.48	0.004
Cushing QoL score	7.13	0.002
Trail Making Test Part A – Total time to complete test (sec)	-4.13	0.003
Trail Making Test Part B – Total time to complete test (sec)	-24.69	< 0.001

p-values for the mean change from baseline to last observation are from the Wilcoxon signed-rank test.

a A significant difference was observed in the high-dose treatment group (mean change -3.2, p = 0.033).

ALT, alanine aminotransferase; aPTT, activated partial thromboplastin time; AST, aspartate aminotransferase; AUC, area under the curve; BDI, Beck Depression Inventory; HOMA-IR, homeostatic model assessment for insulin resistance; QoL, quality of life.

### Hormone Changes

Among all patients with ACTH-dependent CS (n = 26 with values at last visit), the median (min, max) change from baseline to last observed visit was 3.7 (-15.6, 20.0) pmol/L [16.8 (-70.9, 90.9) pg/mL] (*p* = 0.003) for plasma ACTH; 4.0 (-1650.5, 1404.8) nmol/d (1.4 [-598.0, 509.0] µg/24 h) (*p* = 0.873) for 24-h UFC; -0.33 (-30.6, 47.7) nmol/L (-0.01 [-1.1, 1.7] µg/dL) (*p* = 0.815) for LNSC; and 22.0 (-127.0, 339.0) nmol/L (0.8 [-4.6, 12.3] µg/dL) (*p* = 0.239) for serum cortisol.

In the adrenal subgroup (n = 7), median (min, max) change from baseline to last observed visit was 0.2 (0, 1.4) pmol/L (0.9 [0, 6.4] pg/mL) for plasma ACTH; 7.0 (-1662.9, 42.8) nmol/d (2.6 [-602.5, 15.5] µg/24 h) for 24-h UFC; -0.7 (-6.8, 11.8) nmol/L (-0.03 [-0.2, 0.4] µg/dL) for LNSC; and -3.9 (-179.0, 130.0) nmol/L (-0.1 [-6.5, 4.7] µg/dL) for serum cortisol. There were too few patients in the adrenal subgroup to adequately perform statistical testing.

### Safety

Overall, TEAEs were reported in 94.3% of patients (33/35) during treatment with relacorilant, including 88.2% (15/17) in the low-dose group and 100% (18/18) in the high-dose group (**Table 5**). The most commonly reported TEAEs (≥20%) in both the low-dose and high-dose groups combined were back pain (31.4% [11/35]), headache (25.7% [9/35]), peripheral edema (25.7% [9/35]), nausea (22.9% [8/35]), pain at extremities (22.9% [8/35]), diarrhea (20.0% [7/35]), and dizziness (20.0% [7/35]) (**Table 5**). In the high-dose group, the highest incidence of TEAEs was observed with the initial starting dose of 250 mg (100%, 18/18), when compared with incidences of TEAEs at higher doses later (64.3% [9/14], 76.9% [10/13], and 25.0% [2/8] at 300, 350, and 400 mg, respectively). For musculoskeletal and gastrointestinal TEAEs specifically, the highest incidences also occurred at the 250-mg starting dose (61.1% [11/18] and 38.9% [7/18], respectively).

**Table 5:** TEAEs by preferred term in the safety population.

TEAE, n (%)	Low-dose group (n = 17)	High-dose group (n = 18)	Overall population (n = 35)
Patients reporting ≥1 TEAE	15 (88.2)	18 (100.0)	33 (94.3)
TEAE occurring in ≥20% of either the low-dose or high-dose group			
Back pain	4 (23.5)	7 (38.9)	11 (31.4)
Headache	4 (23.5)	5 (27.8)	9 (25.7)
Peripheral edema	4 (23.5)	5 (27.8)	9 (25.7)
Nausea	3 (17.7)	5 (27.8)	8 (22.9)
Pain in extremity	4 (23.5)	4 (22.2)	8 (22.9)
Diarrhea	4 (23.5)	3 (16.7)	7 (20.0)
Dizziness	3 (17.7)	4 (22.2)	7 (20.0)
Arthralgia	2 (11.8)	4 (22.2)	6 (17.1)
Dyspepsia	1 (5.9)	4 (22.2)	5 (14.3)
Myalgia	1 (5.9)	4 (22.2)	5 (14.3)
Abdominal pain	0	4 (22.2)	4 (11.4)

TEAE, treatment-emergent adverse event.

TEAEs leading to discontinuation were reported in 1 patient (5.9%) in the low-dose group and 8 patients (44.4%) in the high-dose group. TEAEs leading to discontinuation in more than one patient were musculoskeletal in nature (e.g., myopathy, back pain).

Five serious TEAEs were reported in 4 patients (pilonidal cyst, myopathy, polyneuropathy, hypertension, and myocardial infarction). All serious TEAEs occurred in the high-dose group. In 3 patients, the serious TEAEs led to discontinuation (myopathy, polyneuropathy, and myocardial infarction). For the patient who developed a serious TEAE of myopathy, a cerebral spinal fluid analysis revealed findings consistent with Guillain-Barre syndrome based on the low cell count and moderately elevated protein. The serious TEAE of polyneuropathy occurred in a patient with underlying uncontrolled diabetes; nerve conduction studies revealed parasympathetic autonomic neuropathy and moderate sensory hypesthesia consistent with diabetic neuropathy. The myocardial infarction (non-ST segment elevation) event occurred in a patient with a history of coronary artery disease, thromboembolism, pulmonary embolism, and myocardial infarction. Cardiac catheterization showed unchanged coronary lesions compared to previous catheterization studies. During the study, no drug-induced vaginal bleeding or hypokalemia was reported. No patients had a platelet count <100,000/µL during the study, nor were there any bleeding events related to a reduction in platelets.

**Figure 4:**
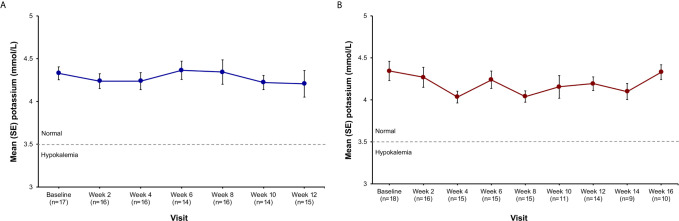
Potassium levels by visit in the **(A)** low-dose group and **(B)** high-dose group.

No clinically significant changes in potassium levels were observed in the study. Mean potassium levels changed from 4.33 mmol/L at baseline to 4.21 mmol/L at week 12 in the low-dose group, and from 4.34 mmol/L at baseline to 4.33 mmol/L at week 16 in the high-dose group (**Figure 4**). Mean changes in potassium levels from baseline to last observed visit across each dose level ranged from -0.12 to 0.02 mmol/L in the low-dose group and from -0.31 to 0.08 mmol/L in the high-dose group.

## Discussion

Relacorilant is a selective glucocorticoid receptor modulator with no affinity for the progesterone receptor (20). In this multicenter, two-step, dose-finding study of patients with endogenous CS and hypertension and/or hyperglycemia, relacorilant improved both clinical hypertension and hyperglycemia parameters in the respective patient groups. Although not formally assessed, differences in response rates between dose groups suggested a dose response. In addition, a number of exploratory endpoints related to cortisol excess were significantly improved.

For patients in the hypertension subgroup, 41.7% (5/12) of the low-dose group and 63.6% (7/11) of the high-dose group met criteria for a clinically meaningful blood pressure response, defined as patients with a decrease of ≥5 mmHg in either mean 24-h SBP or DBP from baseline, and without an increase of concurrent antihypertensive medication or additional antihypertensive medication during the treatment period. In the 24-week SEISMIC study, 38.1% (8/21) of patients met the criteria for hypertension response, which for that trial was defined as a ≥5 mmHg reduction from baseline in DBP assessed as the mean of 2 sequential clinic readings. The response achieved with relacorilant is noteworthy considering that ABPM provides a more accurate representation of blood pressure over a 24-h time period for patients (22, 24). Although the response criteria threshold (≥5 mmHg) appears similar between the studies, they are not equivalent. Average clinic assessments by trained staff are 10/5 mmHg (SBP/DBP) higher than 24-h ABPM (31), and treatment-associated mean 24-h AMBP reductions are disproportionately less than office blood pressure reductions (22, 32). Thus, these phase 2 data suggest a potential benefit for relacorilant in improving hypertension in patients with CS.

For patients in the hyperglycemia subgroup, 15.4% (2/13) of the low-dose group and 50.0% (6/12) the high-dose group met the ad-hoc criteria for a clinically meaningful hyperglycemia response, defined as either a decrease in HbA1c of ≥0.5% from baseline or a normalization or improvement by ≥50 mg/dL (≥2.8 mmol/L) of the 2-h glucose value on the oGTT or a decrease in antidiabetic medication. These criteria were selected because each of them have been used in previous studies to demonstrate a clinically meaningful improvement in the hyperglycemia status of a patient (33–37). Initially, responder criteria were selected for the hyperglycemia population, modelled after the SEISMIC trial. In the SEISMIC trial population, the mean baseline HbA1c (7.4%), 2-h postprandial glucose (296.2 mg/dL [16.4 mmol/L]; data on file), and fasting glucose (149.0 mg/dL [8.3 mmol/L]) were considerably higher than in this trial population (mean baseline HbA1c 6.6%, mean 2-h postprandial glucose 229.4 mg/dL [12.7 mmol/L], mean fasting glucose 119.3 mg/dL [6.6 mmol/L]). In SEISMIC, to qualify as a responder, a ≥25% decrease in AUC_glucose_ on the oGTT without increase or addition of new antidiabetic medication was required. Although the AUC standard would have set an unsafely low glycemic goal in some individual cases in the less severely hyperglycemic population of this study, 12% (3/25) of the pooled hyperglycemia population achieved a ≥25% decrease in AUC_glucose_ from baseline, and more importantly, a significant decrease in AUC_glucose_ from baseline was observed overall. Fructosamine is another biomarker for glucose control and has been shown to correlate closely with HbA1c and glucose levels in clinical trials (26). In an exploratory analysis, significant improvement in serum fructosamine levels was observed in the combined hyperglycemia population, further corroborating the clinical improvements in hyperglycemia with relacorilant noted when applying the ad-hoc responder criteria.

Significant improvement in several additional exploratory endpoints were noted in the pooled patient population. Serum osteocalcin, a marker of bone formation, has been shown to negatively correlate with serum cortisol (25). In this study, relacorilant was associated with a significant increase in serum osteocalcin levels during treatment, indicating increased bone turnover. Nonalcoholic fatty liver disease (NAFLD) is frequently observed in patients with endogenous hypercortisolism (5). In a previous case report, mifepristone treatment was associated with improvement of NAFLD based on a marked reduction in liver enzymes in a patient with hypercortisolism due to an adrenal adenoma (38). Although formal assessments of hepatic steatosis were not performed in this study, relacorilant was associated with significant decreases in both alanine aminotransferase and aspartate aminotransferase values. Significant improvement was also noted in several coagulation parameters (Factor VIII, aPTT, and platelets), cognition, depression, and QoL. These parameters are being further examined in larger, randomized, placebo-controlled phase 3 studies (clinicaltrials.gov NCT03697109, NCT04308590).

The most commonly associated TEAEs with relacorilant were primarily musculoskeletal, gastrointestinal, or nervous system-related in nature. Relacorilant was generally better tolerated when patients were started at 100 mg in the low-dose group. In the high-dose group, with a starting dose of 250 mg, a higher rate of TEAEs and premature discontinuations were observed, with the highest incidence of TEAEs observed early on in the trial at the starting dose of 250 mg, followed by lower incidences on continued treatment at the higher dose levels. This was particularly apparent for musculoskeletal and gastrointestinal TEAEs, suggesting that the higher starting dose prompted symptoms consistent with rapid withdrawal from excess cortisol. Symptoms of cortisol withdrawal (e.g., nausea, fatigue, arthralgia, headache) have also been observed in patients treated with mifepristone (16, 19) and have been shown to last for several weeks (19).

During treatment with the GR antagonist mifepristone, substantial increases in ACTH and cortisol were noted (16, 17). In SEISMIC, UFC levels increased 7.7-fold, and 63% of all patients had at least a 2-fold increase in ACTH. In the present study, the respective median changes from baseline in UFC and ACTH were 4.0 nmol/d (1.4 µg/24 h) and 3.7 pmol/L (16.8 pg/mL) in the ACTH-dependent CS group and 7.0 nmol/d (2.6 µg/24 h) and 0.2 pmol/L (0.9 pg/mL) in the adrenal group. The lower increases in ACTH and cortisol levels observed with relacorilant from this study were consistent with findings from studies conducted in healthy volunteers (39). Administration of relacorilant to healthy subjects was shown to reverse the effects of high-dose prednisone (25 mg) (39) and was not associated with increases in ACTH (data on file). This may reflect differences in the degree of GR antagonism in different tissues at the assessed dosages. Although relacorilant is a potent antagonist of the GR receptor (39), its effects in different tissues (e.g., pituitary) (40) appear to differ from mifepristone (16). Two patients with Cushing disease due to a macroadenoma were enrolled in the phase 2 study and received *de novo* treatment with relacorilant for 12 weeks as preoperative management (40). During treatment, their ACTH levels remained consistent with baseline. Two weeks after their last dose of relacorilant, presurgical imaging revealed a reduction in the size of their tumors (40). *In vitro* studies of relacorilant are underway to further investigate these findings. Compared with patients treated with mifepristone, the noticeably lower increases in UFC and ACTH levels may also explain the absence of drug-induced hypokalemia with relacorilant, and the greater benefit in patients with hypertension (16). Elevated cortisol in CS is associated with activation of the mineralocorticoid receptors upon saturation of the 11β-hydroxysteroid type 2 enzyme receptors, leading to hypokalemia and hypertension (41–43). GR antagonism with mifepristone, leading to a further increase in cortisol levels, can exacerbate these symptoms (19). In fact, hypokalemia occurred in 44% of patients treated with mifepristone in the 24-week clinical trial of 50 adults with endogenous CS (16). Also, unlike mifepristone, there was no drug-induced vaginal bleeding in the study due to the lack of activity at the progesterone receptor.

This study has a number of limitations. First, it is an open-label, phase 2 dose-finding study with a relatively small sample size (no sample size calculation) and short treatment duration, and there was potential heterogeneity in the dose escalation scheme based on the investigators’ clinical judgment. As part of the study design, the low- and high-dose treatment groups were different durations and were performed sequentially, not in parallel, limiting the conclusions regarding a dose response. Also, because of the small sample size, select data from patients with major protocol deviations were included in the analyses of clinical response. Finally, an ad-hoc analysis of hyperglycemia response had to be conducted based on the patient population enrolled; many of the outcome assessments were exploratory in nature; and no adjustments for multiple comparisons were performed. Nonetheless, these phase 2 results provide the first clinical evidence to suggest that relacorilant may offer the clinical benefit of potent and highly specific glucocorticoid modulation in patients with endogenous CS without the undesirable effects mediated by mifepristone’s activity at the progesterone receptor or its frequent mineralocorticoid activation *via* its marked elevation of cortisol levels. Increases in ACTH and cortisol with relacorilant were substantially less than with mifepristone, which may greatly benefit patients.

Two larger phase 3 studies to further investigate the clinical efficacy and safety of relacorilant in patients with endogenous CS and hypertension or IGT/T2DM and in patients with CS caused by a cortisol-producing adrenal adenoma or bilateral hyperplasia are in progress. The safety data from this phase 2 study suggest that patients should begin relacorilant treatment at a relatively low dose. Because the starting dose of 100 mg in the low-dose group was well tolerated, it was chosen as the starting dose in the phase 3 trials of relacorilant. In those trials, patients begin treatment at 100 mg/d for 2 weeks and the dose is gradually escalated, enhancing tolerability and treatment persistence. Further examination of the tissue specificity of relacorilant is also underway, including the effect of relacorilant on pituitary size in the phase 3 study. *In vitro* studies of relacorilant are being conducted to further investigate the different effects of relacorilant and mifepristone on cortisol and somatostatin receptor expression.

## Data Availability Statement

The datasets presented in this study can be found in online repositories. The names of the repository/repositories and accession number(s) can be found below: Grouped datasets analyzed during the current study are available here. Additional individual datasets generated during and/or analyzed are not publicly available but are available from the corresponding author on reasonable request.

## Ethics Statement

The studies involving human participants were reviewed and approved by the institutional review board at each study center. The patients/participants provided their written informed consent to participate in this study.

## Author Contributions

Study concept and design: AM. Study investigators who provided study materials and/or patients: RP, IB, RF, AK, JK, MG, CM, and MT. Statistical analysis: NE. Analyzed and interpreted clinical data: All authors. Wrote manuscript or critically revised it for content: All authors. All authors contributed to the article and approved the submitted version.

## Funding

This study was funded by Corcept Therapeutics. Open Access publication fees were paid by Corcept Therapeutics.

## Conflict of Interest

The authors declare that this study received funding from Corcept Therapeutics (Menlo Park, CA, USA). The funder had a role in study design, data collection and analysis, and AM, as an author of the manuscript and employee of Corcept Therapeutics, had a role in the study design, the decision to publish, the interpretation of clinical data, the revision of the manuscript, and approval of the final manuscript to submit. Open Access publication fees were paid by Corcept Therapeutics. **RP**: Consultant: Ferring, Ipsen, Novartis, Pfizer, ViroPharma-Shire; Speaker: Novartis, ViroPharma-Shire; Research support: Corcept Therapeutics, Novartis, ViroPharma-Shire; Grant support: IBSA, Novartis, Pfizer, ViroPharma-Shire. **IB**: Consultant: HRA Pharma, Sparrow Pharmaceutics, Strongbridge; Data and Safety Monitoring Panel, Adrenas. **RF**: Consultant: Corcept Therapeutics; Speaker: HRA Pharma. **AK**: Consultant: Strongbridge; Research support: Corcept Therapeutics. **MG**: Research support: Corcept Therapeutics, Crinetics, Ionis, Ipsen, Novartis, Novo Nordisk, Opko, Strongbridge, Teva. **CM**: Consultant: Horizon Therapeutics; Research support: Corcept Therapeutics, Eli Lilly, Medtronic. **MT**: Consultant: HRA Pharma; Research support: Corcept Therapeutics. **NE**: Consultant: Corcept Therapeutics, Pentara, Trialwise. **AM**: Employee: Corcept Therapeutics.

The remaining author (JK) declares that the research was conducted in the absence of any commercial or financial relationships that could be construed as a potential conflict of interest.

The reviewer AALC declared a shared affiliation with one of the authors, RP, to the handling editor at time of review.

Author NE was employed by company Trialwise.

## Abbreviations

ABPM, ambulatory blood pressure monitoring; ACTH, adrenocorticotropic hormone; ALT, alanine aminotransferase; aPTT, activated partial thromboplastin time; AST, aspartate aminotransferase; AUC, area under the curve; BDI-II, Beck Depression Inventory; BMI, body mass index; CD, Cushing disease; CI, confidence interval; CS, Cushing syndrome; DBP, diastolic blood pressure; DST, dexamethasone suppression test; ET, early termination; GR, glucocorticoid receptor; HbA1c, glycated hemoglobin; HOMA-IR, homeostatic model assessment of insulin resistance; IGT, impaired glucose tolerance; LNSC, late-night salivary cortisol; NAFLD, nonalcoholic fatty liver disease; oGTT, oral glucose tolerance test; QoL, quality of life; SBP, systolic blood pressure; SD, standard deviation; SGRM, selective glucocorticoid receptor modulator; TEAE, treatment-emergent adverse event; T2DM, type 2 diabetes mellitus; UFC, urinary free cortisol; ULN, upper limit of normal.
