# Corrigendum: Relacorilant, a Selective Glucocorticoid Receptor Modulator, Induces Clinical Improvements in Patients With Cushing Syndrome: Results From A Prospective, Open-Label Phase 2 Study

**DOI:** 10.3389/fendo.2022.899616

**Published:** 2022-04-27

**Authors:** Rosario Pivonello, Irina Bancos, Richard A. Feelders, Atil Y. Kargi, Janice M. Kerr, Murray B. Gordon, Cary N. Mariash, Massimo Terzolo, Noel Ellison, Andreas G. Moraitis

**Affiliations:** ^1^ Dipartimento di Medicina Clinica e Chirurgia, Sezione di Endocrinologia, Università Federico II di Napoli, Naples, Italy; ^2^ Department of Internal Medicine, Division of Endocrinology, Diabetes, Metabolism, and Nutrition, Mayo Clinic, Rochester, MN, United States; ^3^ Department of Internal Medicine, Division of Endocrinology, Erasmus Medical Center, Rotterdam, Netherlands; ^4^ Department of Medicine, Division of Endocrinology, Diabetes and Metabolism, University of Miami, Miami, FL, United States; ^5^ Department of Endocrinology, Division of Endocrinology, Metabolism and Diabetes, University of Colorado Denver, Aurora, CO, United States; ^6^ Allegheny Neuroendocrinology Center, Allegheny General Hospital, Pittsburgh, PA, United States; ^7^ Methodist Research Institute, Indiana University School of Medicine, Indianapolis, IN, United States; ^8^ Department of Clinical and Biological Sciences, Internal Medicine 1 − San Luigi Gonzaga Hospital, University of Turin, Orbassano, Italy; ^9^ Trialwise, Inc, Houston, TX, United States; ^10^ Corcept Therapeutics, Menlo Park, CA, United States

**Keywords:** clinical trial, cortisol, Cushing syndrome, glucocorticoid, hypercortisolism, hyperglycemia, hypertension, relacorilant

In the original article, there was an error. In the ACTH-dependent CS group, the median (min) change in ACTH from baseline to last observed visit in conventional units (pg/mL) should be 16.8 (-70.9) instead of 0.8 (-3.4).

A correction has been made to **Results,** *“Hormone Changes,”* paragraph 1:

“Among all patients with ACTH-dependent CS (n = 26 with values at last visit), the median (min, max) change from baseline to last observed visit was 3.7 (-15.6, 20.0) pmol/L [16.8 (-70.9, 90.9) pg/mL] (p = 0.003) for plasma ACTH…”

A correction has been made to **Discussion,** paragraph 6:

“In the present study, the respective median changes from baseline in UFC and ACTH were 4.0 nmol/d (1.4 μg/24 h) and 3.7 pmol/L (16.8 pg/mL) in the ACTH-dependent CS group…”

In the original article, there was an error. A negative sign was omitted from the min LNSC value in conventional units (µg/dL). Instead of 1.1, it should be -1.1 µg/dL.

A correction has been made to **Results,** *“Hormone Changes,”* paragraph 1:

“… -0.33 (-30.6, 47.7) nmol/L [-0.01 (-1.1, 1.7) µg/dL] (p = 0.815) for LNSC…”

The authors apologize for the errors and state that this does not change the scientific conclusions of the article in any way. The original article has been updated.

## Publisher’s Note

All claims expressed in this article are solely those of the authors and do not necessarily represent those of their affiliated organizations, or those of the publisher, the editors and the reviewers. Any product that may be evaluated in this article, or claim that may be made by its manufacturer, is not guaranteed or endorsed by the publisher.

